# Transient Cytopenias as a Rare Presentation of Classic Galactosemia

**DOI:** 10.7759/cureus.23101

**Published:** 2022-03-12

**Authors:** Maria Gianniki, Irini Nikaina, Georgia Avgerinou, Christina Kanaka-Gantenbein, Tania Siahanidou

**Affiliations:** 1 First Department of Pediatrics, National and Kapodistrian University of Athens, Athens, GRC

**Keywords:** liver failure, thrombocytopenia, cytopenias, galactosemia, newborn

## Abstract

Although galactosemia can be detected through neonatal screening, some cases are characterized by rapid and severe presentation before screening results become available. We report the case of a neonate with classic galactosemia presenting with acute liver failure and cytopenias (thrombocytopenia, anemia, and neutropenia). Neonatal screening results showed increased galactose and phenylalanine levels. The diagnosis of galactosemia was confirmed by the measurement of galactose-1-phosphate uridyltransferase (GALT) activity in erythrocytes. Two mutations of the *GALT* gene (c.563 A>G [p. Q188R] and c.957C>A [p.H319Q]) were revealed. High clinical suspicion of galactosemia is crucial to identify, as early as possible, cases with classical or even unusual presentation, and to initiate early treatment that could change the disease course and improve outcomes. Cytopenias should be included in the broad phenotypic spectrum of galactosemia.

## Introduction

Classic galactosemia is a rare (one case per 40,000-60,000 newborns) autosomal recessive inborn error of galactose metabolism caused by galactose-1-phosphate uridyltransferase (GALT) deficiency [1]. The majority of the affected infants are born phenotypically healthy but experience a rapid and severe deterioration following milk exposure due to a toxic build-up of intermediates of the galactose metabolism (Leloir) pathway, such as galactose-1-phosphate (gal-1-p) and galactitol, because of mutations in the *GALT *gene [1,2]. Galactosemia should be urgently diagnosed because it can lead even to death in the neonatal period if left untreated. We report the case of a neonate with classic galactosemia that presented as acute liver failure and hyperammonemia, as well as transient cytopenias, which have very rarely been described in the literature [3].

## Case presentation

A seven-day-old female neonate born at 38 gestational weeks, after an uncomplicated pregnancy, was admitted due to poor feeding, vomiting, weight loss, and jaundice. There was no parental consanguinity or family history of an inherited disorder. At birth, the baby’s body weight (3,110 g), length (49 cm), and head circumference (34 cm) were normal (25th-50th percentile). Apgar score was 9 and 10 in the first and fifth minutes, respectively. She started breastfeeding immediately after birth, and three days later the mother and infant were discharged from the maternity hospital.

On admission, hypotonia, dehydration, hepatomegaly (liver at 4 cm below costal margin), and increased bilirubin (total 12.8 mg/dL; conjugated 1.7 mg/dL), aspartate transaminase (135 U/L; normal 10−60), alanine transaminase (244 U/L; normal 5−45), and alkaline phosphatase (1105 U/L; normal 60−240) serum levels were noticed. In the next two days, the neonate deteriorated with the rapid development of lethargy, an episode of convulsions treated with phenobarbital, and petechiae and bleeding at venipuncture sites. Hyperammonemia (248 μg/dL; normal <80) and abnormal hemostasis screening [international normalized ratio 4.2; prothrombin time 61.7 seconds (normal 10-14); activated partial thromboplastin time 111.6 seconds (normal 20-39)] were recorded. A septic screen was repeatedly negative. Increased methionine (106 μmol/L; normal 6-36) and phenylalanine plasma levels (644 μmol/L; normal 16-71), and an abnormal transferrin isoelectric focusing (TfIEF) pattern, as in congenital disorders of glycosylation, were detected. The results of the newborn screening showed increased galactose (>50 mg/dL; normal <12) and phenylalanine levels (529 μmol/L; normal <120); findings were suggestive of galactosemia. Ophthalmologic examination showed a bilateral “oil drop” cataract. The diagnosis of galactosemia was confirmed by the measurement of GALT activity in erythrocytes (2.3 U/g hemoglobulin, normal >3.5). Genetic testing revealed the presence of two mutations of the *GALT *gene as compound heterozygous status, namely, c.563 A>G [p.Q188R] and c.957C>A [p.H319Q].

**Figure 1:**
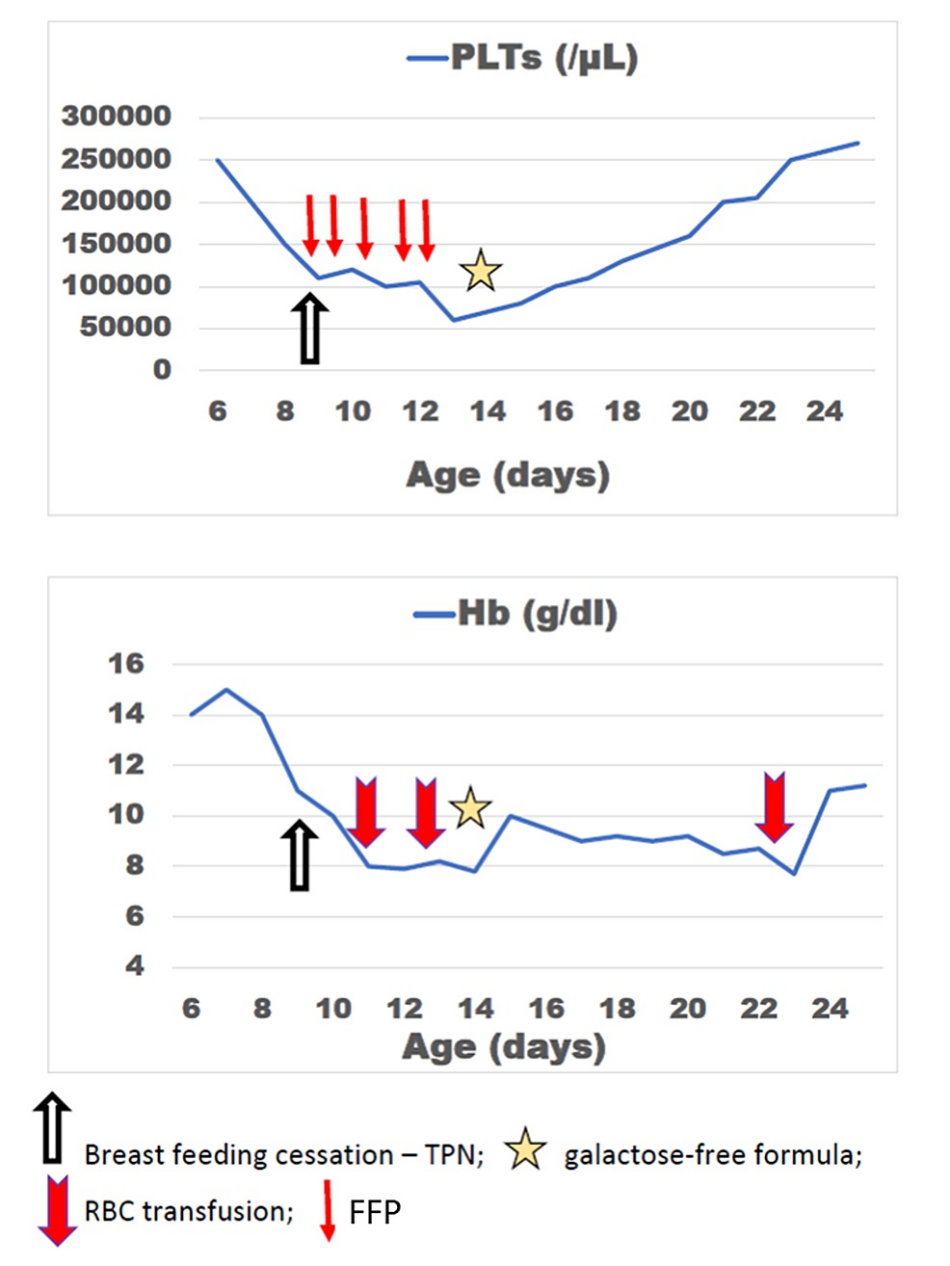
Platelet counts and hemoglobin levels during hospitalization. PLTs: platelets; Hb: hemoglobin; RBC: red blood cells; FFP: fresh frozen plasma; TPN: total parenteral nutrition

During the first few days of hospitalization, the neonate also presented anemia (hemoglobin 7 g/dL), with low reticulocytes (0.89%), decreased white blood cell count (minimum 4,730/mm^3^), neutropenia (minimum neutrophil count 870/mm^3^), and thrombocytopenia (minimum platelets 65,000/mm^3^) (Figure 1).

**Figure 2:**
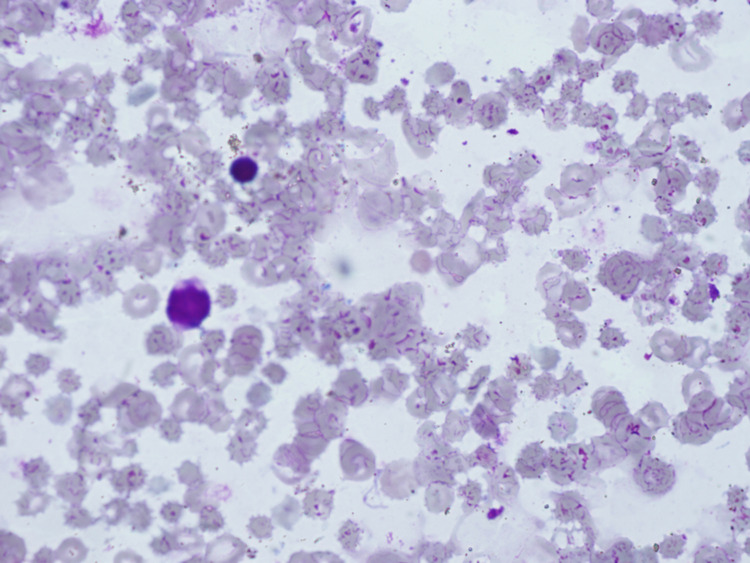
Bone marrow aspirate showing cellularity less than 25% (Giemsa stain; Nikon plan, 10×0.25).

With the suspicion of secondary hemophagocytosis, serum ferritin (1,750 μg/dl; normal 10-150) and triglyceride levels (67 mg/dL, normal 30-130) were assessed, and a bone marrow aspiration was performed. Bone marrow was hypoplastic, whereas no hemophagocytosis was observed (Figure 2).

The patient did not fulfill the diagnostic criteria for hemophagocytic lymphohistiocytosis [4]. Treatment was symptomatic including phototherapy for jaundice, sodium benzoate (250 mg/kg/day), and phenylbutyrate (250 mg/kg/day) for hyperammonemia, and red blood cell and fresh frozen plasma transfusions. Breastfeeding was discontinued and the baby was fed initially with total parenteral nutrition changed to a galactose-free formula following the diagnosis. A few days later, her clinical signs and laboratory parameters improved considerably and the neonate started gaining weight. Plasma amino acid profile, liver function tests, and the abnormal TfIEF patterns were normalized.

## Discussion

This is a case of galactosemia in a neonate who presented with both acute liver failure and cytopenias. Neonatal acute liver failure has been well described in the literature in patients with galactosemia; on the contrary, cytopenias have only rarely been reported either in the context of secondary hemophagocytic lymphohistiocytosis in galactosemia [3], similar to other metabolic disorders [5,6] or terms of inherited/chronic thrombocytopenia or pancytopenia associated with mutation of UDP-galactose-4-epimerase [7,8]. Hemophagocytic lymphohistiocytosis was not confirmed in our patient because only three out of the eight diagnostic criteria [4] were fulfilled (cytopenia, low fibrinogen, and elevated ferritin). Furthermore, neonatal hemochromatosis/gestational alloimmune liver disease presenting with acute liver failure and severe thrombocytopenia could not be ruled out; nevertheless, no liver biopsy or imaging study was performed considering the patient’s recovery and the established galactosemia diagnosis. Interestingly, the bone marrow of our patient was found hypoplastic; moreover, an abnormal TfIEF pattern was observed. Previous studies have shown that proper glycosylation is crucial to normal hemopoiesis, in particular to megakaryocyte and platelet development; defects in genes regulating sugar metabolism and glycosylation can be associated with bone marrow failure [7,8]. Moreover, in an animal model of galactosemia, newborn *GALT *gene-trapped mice presented manifestations of oxidative stress in red blood cells (altered glutathione/oxidized glutathione ratio), a fact to which our patient’s anemia could be also attributed [2]. The normalization of both cytopenias and TfIEF pattern in our patient a few days after diet intervention further supports the notion that these abnormalities consisted of secondary toxic consequences of metabolic injury. In patients with galactosemia, the evaluation of the TfIEF pattern has been proposed as a useful indicator of compliance with galactose restriction [9].

As mentioned, the neonatal metabolic screening (Guthrie test) in our patient showed both increased galactose and phenylalanine levels. Such findings necessitate careful interpretation so as not to misdiagnose a condition characterized by secondary hypergalactosemia, such as congenital hepatitis, congenital hepatic arteriovenous malformations, type I tyrosinemia, type II citrullinemia, or other metabolic disorders with hepatocellular disease and Fanconi-Bickel syndrome. On the other hand, elevated phenylalanine levels on newborn screening may be a secondary effect of liver dysfunction in cases with galactosemia [10].

## Conclusions

In many developed countries, including Greece, the diagnosis of galactosemia is made early by newborn screening programs. Unfortunately, there are some cases in which signs and symptoms appear early, even before the screening test is performed, or before its results are available, as was the case in our patient. As early diagnosis and early treatment can change the disease course, a high clinical suspicion is crucial to identify as early as possible cases of galactosemia with classical or unusual presentation. Cytopenias, especially thrombocytopenia, even transient, should be included in the broad phenotypic spectrum of galactosemia.
